# An artificial intelligence system to predict the optimal timing for mechanical ventilation weaning for intensive care unit patients: A two-stage prediction approach

**DOI:** 10.3389/fmed.2022.935366

**Published:** 2022-11-18

**Authors:** Chung-Feng Liu, Chao-Ming Hung, Shian-Chin Ko, Kuo-Chen Cheng, Chien-Ming Chao, Mei-I Sung, Shu-Chen Hsing, Jhi-Joung Wang, Chia-Jung Chen, Chih-Cheng Lai, Chin-Ming Chen, Chong-Chi Chiu

**Affiliations:** ^1^Department of Medical Research, Chi Mei Medical Center, Tainan, Taiwan; ^2^Department of General Surgery, E-Da Cancer Hospital, Kaohsiung, Taiwan; ^3^College of Medicine, I-Shou University, Kaohsiung, Taiwan; ^4^Department of Respiratory Therapy, Chi Mei Medical Center, Tainan, Taiwan; ^5^Department of Internal Medicine, Chi Mei Medical Center, Tainan, Taiwan; ^6^Department of Intensive Care Medicine, Chi Mei Medical Center, Liouying, Taiwan; ^7^Department of Dental Laboratory Technology, Min-Hwei College of Health Care Management, Liouying, Taiwan; ^8^Department of Anesthesiology, Chi Mei Medical Center, Tainan, Taiwan; ^9^Department of Anesthesiology, National Defense Medical Center, Taipei, Taiwan; ^10^Department of Information Systems, Chi Mei Medical Center, Tainan, Taiwan; ^11^Division of Hospital Medicine, Department of Internal Medicine, Chi Mei Medical Center, Tainan, Taiwan; ^12^Department of Intensive Care Medicine, Chi Mei Medical Center, Tainan, Taiwan; ^13^School of Medicine, College of Medicine, I-Shou University, Kaohsiung, Taiwan; ^14^Department of Medical Education and Research, E-Da Cancer Hospital, Kaohsiung, Taiwan; ^15^Department of General Surgery, Chi Mei Medical Center, Tainan, Taiwan

**Keywords:** artificial intelligence, machine learning, intensive care unit, weaning mechanical ventilation, optimal weaning timing

## Abstract

**Background:**

For the intensivists, accurate assessment of the ideal timing for successful weaning from the mechanical ventilation (MV) in the intensive care unit (ICU) is very challenging.

**Purpose:**

Using artificial intelligence (AI) approach to build two-stage predictive models, namely, the try-weaning stage and weaning MV stage to determine the optimal timing of weaning from MV for ICU intubated patients, and implement into practice for assisting clinical decision making.

**Methods:**

AI and machine learning (ML) technologies were used to establish the predictive models in the stages. Each stage comprised 11 prediction time points with 11 prediction models. Twenty-five features were used for the first-stage models while 20 features were used for the second-stage models. The optimal models for each time point were selected for further practical implementation in a digital dashboard style. Seven machine learning algorithms including Logistic Regression (LR), Random Forest (RF), Support Vector Machines (SVM), K Nearest Neighbor (KNN), lightGBM, XGBoost, and Multilayer Perception (MLP) were used. The electronic medical records of the intubated ICU patients of Chi Mei Medical Center (CMMC) from 2016 to 2019 were included for modeling. Models with the highest area under the receiver operating characteristic curve (AUC) were regarded as optimal models and used to develop the prediction system accordingly.

**Results:**

A total of 5,873 cases were included in machine learning modeling for Stage 1 with the AUCs of optimal models ranging from 0.843 to 0.953. Further, 4,172 cases were included for Stage 2 with the AUCs of optimal models ranging from 0.889 to 0.944. A prediction system (dashboard) with the optimal models of the two stages was developed and deployed in the ICU setting. Respiratory care members expressed high recognition of the AI dashboard assisting ventilator weaning decisions. Also, the impact analysis of with- and without-AI assistance revealed that our AI models could shorten the patients’ intubation time by 21 hours, besides gaining the benefit of substantial consistency between these two decision-making strategies.

**Conclusion:**

We noticed that the two-stage AI prediction models could effectively and precisely predict the optimal timing to wean intubated patients in the ICU from ventilator use. This could reduce patient discomfort, improve medical quality, and lower medical costs. This AI-assisted prediction system is beneficial for clinicians to cope with a high demand for ventilators during the COVID-19 pandemic.

## Introduction

Mechanical ventilation (MV) is frequently applied in the intensive care unit (ICU). Approximately eight hundred thousand patients receive MV annually in the United States (1). Extubation decision is critical during an ICU stay. An early trial of the weaning process and successful extubation may lower the medical costs and ventilator-related complication rates. Besides, it could improve the patient’s prognosis (2–4). Therefore, after the recovery of the critical illness, clinicians should immediately prepare to liberate the patients from MV. Evaluation of an ICU patient’s fitness for weaning and subsequent extubation is objectively referred to the airway, respiratory, neurological parameters, *etc.* (5). Most of the times, liberation from MV requires three steps – readiness testing, weaning, and extubating and the process of MV liberation is dynamic and complicated. In daily practice, extubation is usually left to the discretion of the clinician (6); therefore, various protocols for ventilator weaning have been established and assessed to increase the extubation rate (7–18).

Despite following the recommended extubation process established in the American Thoracic Surgery weaning protocol, the failure rate still ranges from 10 to 15% of ICU patients in the United States (19). Truthfully, there has been no significant decrease in extubation failure in the past decades. Therefore, an advanced strategy is mandatory to increase the prediction accuracy (20). Several multivariate outcome prediction models have evolved in many aspects of health care research in these years. They include artificial neural networks (ANN), logistic regression (LR) models, random forest (RF) models, and support vector machines (SVM) (21–26). Machine learning (ML) is a subject of computer science that incorporates numerous components to empower the systems to learn from currently acquired data, predict the outcome, and make changes in action when faced with a new problem. Clinically, ML could increase the prediction rate of successful weaning from ventilatory support. The parameters considered in the prediction of successful weaning and extubation were based on literature (27–34) and clinical experience.

Many studies (35–38) have reported the usefulness of AI in the ICU, such as the early warning systems that predict the risk of physiological deterioration in acutely ill patients, the development of acute respiratory distress syndrome, the early development of sepsis and the pathogen that causes it, and clinical outcome and mortality. However, studies on the utility of AI in predicting the weaning and extubation process among critically ill patients requiring MV are limited (39–43), while those that explore AI’s capacity to predict the weaning timing for intubated patients are rare.

This study aims to develop an AI digital dashboard to remind the ICU clinicians of the optimal timing for weaning initiation, propose an individualized treatment recommendation, and assist in making extubation decisions. Data that can be conveniently collected were chosen as variables for building the prediction model, including patients’ characteristics and respiratory pattern parameters during spontaneous breathing trials (SBTs). A preliminary impact analysis was performed after AI assistance to predict successful extubation in ICU patients.

## Materials and methods

### Ethical consideration

This study was reviewed and accepted by the CMMC (IRB Serial No.: 10912-016). The process was performed according to the approved guidelines and regulations, and informed consent was waived from the patients because of the nature of our retrospective study.

### Study design

**FIGURE 1:**
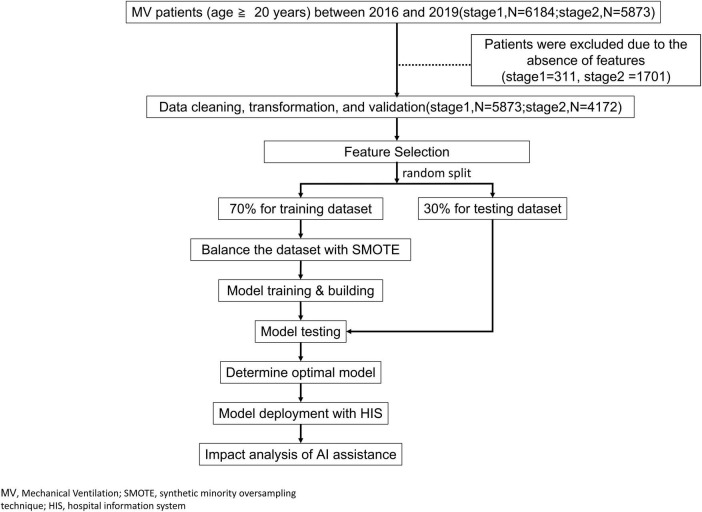
Study flow.

**FIGURE 2:**
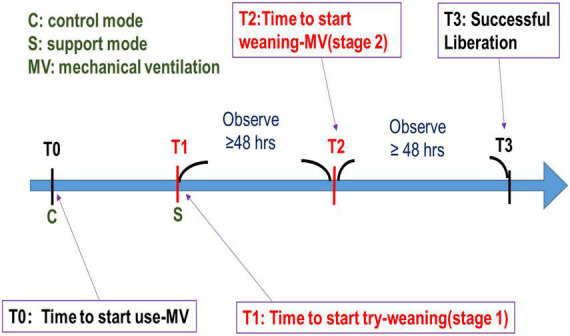
Two-stage weaning assessment.

The study flow chart is demonstrated in Figure 1. In the beginning, we established a professional team, including clinicians, respiratory therapists, data scientists, and information technology engineers, and held regular meetings and discussions. We retrospectively collected data from adult ventilated patients (≥20 years old) who stayed at the ICU of CMMC from January 2016 to December 2019. Patients who signed the DNR (Do not resuscitate) were excluded. According to clinical experience, if the try-weaning timing is appropriate, the success probability of the final complete weaning ventilator will also increase. Therefore, this study divided the complete assessment of ventilator use into two stages: (1) the try-weaning stage and (2) the complete weaning MV stage (Figure 2).

Try-weaning stage means switching the ventilator from control mode to support mode for an ICU patient, while the complete weaning MV stage means transitioning from support mode ventilation to oxygen therapy or extubation for an ICU patient.

### Setting and data source

Chi Mei Medical Center is a large hospital in Tainan, Taiwan with 1288 beds, including 109 ICU beds. It has a comprehensive hospital information system to store each kind of clinical data such as demographics, diagnoses, vital signs, laboratory data, and prescribed medications in the database. Since 2016, CMMC adopted IoT technology to capture parameters from the MV in ICUs automatically per minute. So far, big data from MV was cumulated and ready for further AI and machine learning study.

### Features and outcome variables

The first stage model used 25 features, including primary patient data of age, Acute Physiology and Chronic Health Evaluation II (APACHE II) score, Therapeutic Intervention Scoring System (TISS) score, and the first and last Internet of Thing (IoT) data of the respirator consisting of inspired oxygen fraction (FiO_2_), positive end-expiratory pressure (PEEP), respiratory rate (RR), minute ventilation (Mv), peak inspiratory pressure (Ppeak), mean airway pressure (mPaw), peripheral oxygen saturation (SpO_2_), expiratory tidal volume (Vte), heart rate (HR), systolic blood pressure (SBP), and diastolic blood pressure (DBP). Based on clinical experience, the outcome variable was binary coded with 1 (i.e., successful try-weaning), which means that MV was shifted from the control mode to the support mode for at least 48 h, otherwise it was coded with 0.

The second stage model used 20 features, including primary data consisting of age, APACHE II score, and TISS score; and the last respirator IoT data before extubation consisting of FiO_2_, PEEP, RR, Mv, Ppeak, mPaw, SpO_2_, pressure support level (PSL), tidal volume with pressure support (PSLvolume), body temperature (BT), HR, SBP, DBP, Glasgow Coma Scale eye-opening (GCS_E), GCS motor response (GCS_M), SBT count during support mode, and sputum suction count within 24 hours before extubation (Suction). The outcome variable was binary coded with 1 (i.e., successful weaning MV), which means weaning from MV for at least 48 h, otherwise coded with 0. This is also accepted as a basis for the provision of government-related health subsidies in Taiwan.

All potential features were selected based on the literature (6, 7, 44, 45), clinic availability and the experience of clinicians. We performed correlation analysis between features and outcomes to assist in feature selection decisions. Features with the raw data were obtained from the hospital information system (HIS) and real-time IoT transferring from ventilators.

### Model building and measurement

Raw data was collected from the electronic medical records of ICU to build the models for stage 1 and stage2. We randomly divided the cleaned data into 70% training and 30% testing data. Due to the data imbalance problem (fewer cases in the minority class), we applied the Synthetic minority over-sampling technique (SMOTE) method to process the training data (46). We performed a grid search for five-fold cross-validation on the training dataset to obtain the best hyper-parameters for modeling. Finally, we used the testing dataset (also called hold-out dataset) for the final evaluation of the model quality. Four model quality indicators of accuracy, sensitivity, specificity, and AUC (area under the ROC) were applied to assess the model quality. However, the overall model performance is generally evaluated by AUC in many medical studies since both true/false positive and true/false negative are fairly considered. Thus, AUC was used in this study as the main indicator to determine the optimal model. We used the optimal models for subsequent implementation of the predictive system.

Each outcome used a variety of ML algorithms to build models, including LR, RF, SVM, K Nearest Neighbor (KNN), lightGBM, XGBoost, and Multilayer Perception (MLP). The ML models were performed based on Sklearn library and related ML modules in Python.

The main purpose of this study was to predict the optimal timing to wean MV, not just successful weaning or not; thus, we divided each stage into 11 time periods based on clinical experience, and built 11 prediction models with the period data rather than building a single model with the end-point data of ICU patients with MV. That is, it is of great value to predict whether a patient can successfully wean or not over time. After all, most patients with MV in ICU will eventually be successfully weaned but we expect timely or even early safe weaning of MV to avoid overuse rather than just predicting success or not (in CMMC, the average extubation success rate exceeds 85%). However, hospitals can reduce or increase the predictive periods according to their needs while implementing.

Stage 1 of timing prediction for successful try-weaning involves the following: After the patient enters the ICU for intubation, we built 11 models for 11 prediction time points, namely: 8th hour, 12th hour, 24th hour, 36th hour, 48th hour, 60th hour, 72nd hour, 84th hour, 96th hour, 108th hour, and 120th hour. The first stage is considered a success if the MV is shifted from assist control to support mode for at least 48 h.

Stage 2 of timing prediction for successful weaning-MV involves the following: We built 11 models in days (after the patient completed the first stage successfully). The second stage is considered a success if the patient can last longer than 48 h after extubation from the support mode or leave the ICU safely for shorter than 48 h (47).

The data used in each model came from the data collected at this time point. For example, the 60th HR model used data of patients, which was collected at or nearer the 60th hour time point of using the respirator.

### Two-stage artificial intelligence prediction system development of the optimal models

We chose the optimal models for each stage to develop a two-stage prediction system in a digital dashboard style to assist the weaning decision of respiratory medical teams. The system was developed using Microsoft Visual Studio^®^ with VB language.

The web-based dashboard is linked to the real-time database of the existing HIS which could retrieve the required feature values of a specific model. The clinical staff could obtain the related predictive data and figure out the best timing of MV weaning by just previewing the patient’s data in the dashboard. The dashboard automatically retrieves the clinical data of the patient for AI prediction without the need for manual input and immediately displays the probabilities of successful MV weaning at each time from the beginning of ventilator use to the nearest future time point. The dashboard would automatically refresh the prediction for all patients every 60 min.

For example, if a patient has used MV for over 50 hours, the dashboard will show the probability of the 24th, 48th, and 72nd hour. The respiratory care team can further double-click on the targeted patient to prompt a new page to overview the detailed feature values at that predicting period. By monitoring the trend curve of the successful probabilities (in colored balls) and detailed feature values, the respiratory care team could evaluate whether each patient is eligible to start trying weaning or liberate the individual from MV more objectively and efficiently at this time point.

## Results

### Demographics

We retrospectively collected 6,184 cases of patients who used MV in CMMC ICU from 2016/1/1 to 2019/12/31. After excluding the cases with missing values, 5,873 cases were included for modeling in Stage 1 and 4,172 cases were included in Stage 2. Tables 1, 2 show the patients’ demographics in Stage 1 and Stage 2 respectively.

**TABLE 1:** Stage 1 demography.

Feature		Overall	
		*N* = 5873	
Age, mean (SD)		64.0 (15.3)	
APACHE II score, mean (SD)		19.6 (8.5)	
TISS score, mean (SD)		29.7 (7.9)	
IoT data	First*		Last**
FiO_2_, mean (SD)	45.4 (20.8)		32.3 (15.0)
PEEP, mean (SD)	5.6 (1.4)		5.8 (1.5)
RR, mean (SD)	15.8 (4.2)		14.0 (4.0)
Mv, mean (SD)	8.8 (2.8)		7.9 (2.4)
Ppeak, mean (SD)	24.3 (4.3)		23.1 (4.1)
mPaw, mean (SD)	10.6 (2.8)		10.1 (2.7)
SpO_2_, mean (SD)	98.7 (2.3)		98.1 (4.7)
Vte, mean (SD)	576.0 (115.5)		576.8 (117.8)
HR, mean (SD)	93.8 (21.3)		84.4 (21.4)
SBP, mean (SD)	139.4 (37.8)		128.8 (33.1)
DBP, mean (SD)	79.2 (21.6)		68.9 (18.7)
**Outcome**			
Successful try-weaning within 8 h, *n* (%)		1,113 (19.0)	
Successful try-weaning within 12 h, *n* (%)		1,588 (27.0)	
Successful try-weaning within 24 h, *n* (%)		2,840 (48.4)	
Successful try-weaning within 36 h, *n* (%)		3,112 (53.0)	
Successful try-weaning within 48 h, *n* (%)		3,523 (60.0)	
Successful try-weaning within 60 h, *n* (%)		3,710 (63.2)	
Successful try-weaning within 72 h, *n* (%)		3,968 (67.6)	
Successful try-weaning within 84 h, *n* (%)		4,114 (70.0)	
Successful try-weaning within 96 h, *n* (%)		4,281 (72.9)	
Successful try-weaning within 108 h, *n* (%)		4,373 (74.5)	
Successful try-weaning within 120 h, *n* (%)		4,506 (76.7)	

*First: data of the first record in control mode. **Last: data of the last record in control mode. SD, Standard Deviation; IoT, Internet of Things; APACHE II, Acute Physiology and Chronic Health Evaluation II; TISS, Therapeutic intervention scoring system; FiO2, the fraction of inspired oxygen; PEEP, positive end-expiratory pressure; RR, respiratory rate; Mv, minute ventilation; Ppeak, peak inspiratory pressure; mPaw, mean airway pressure; SpO2, peripheral oxygen saturation; Vte, expiratory tidal volume; HR, heart rate; SBP, systolic blood pressure; DBP, diastolic blood pressure.

**TABLE 2:** Stage 2 demography.

Feature	Overall
	*N* = 4172
Age, mean (SD)	64.3 (15.3)
APACHE II score, mean (SD)	18.9 (8.0)
TISS score, mean (SD)	29.6 (7.7)
FiO_2_, mean (SD)	26.1 (2.1)
PEEP, mean (SD)	5.2 (0.7)
RR, mean (SD)	16.4 (5.0)
Mv, mean (SD)	7.7 (2.4)
PSL, mean (SD)	9.4 (2.0)
PSLvolume, mean (SD)	484.4 (125.3)
Ppeak, mean (SD)	15.4 (2.0)
mPaw, mean (SD)	8.3 (1.8)
SpO_2_, mean (SD)	98.7 (1.6)
BT, mean (SD)	36.6 (0.5)
HR, mean (SD)	85.4 (16.7)
SBP, mean (SD)	135.1 (23.8)
DBP, mean (SD)	72.2 (14.9)
GCS_E, mean (SD)	3.5 (0.7)
GCS_M, mean (SD)	5.7 (0.7)
SBT times, mean (SD)	1.4 (2.8)
Suction times, mean (SD)	5.0 (4.4)
**Outcome**	
Successful weaning-MV within 24 h, *n* (%)	1,807 (43.3)
Successful weaning-MV within 48 h, *n* (%)	2,133 (51.1)
Successful weaning-MV within 72 h, *n* (%)	2,451 (58.7)
Successful weaning-MV within 96 h, *n* (%)	2,709 (64.9)
Successful weaning-MV within 120 h, *n* (%)	2,910 (69.8)
Successful weaning-MV within 144 h, *n* (%)	3,070 (73.6)
Successful weaning-MV within 168 h, *n* (%)	3,198 (76.7)
Successful weaning-MV within 192 h, *n* (%)	3,312 (79.4)
Successful weaning-MV within 216 h, *n* (%)	3,402 (81.5)
Successful weaning-MV within 240 h, *n* (%)	3,462 (83.0)
Successful weaning-MV within 264 h, *n* (%)	3,518 (84.3)

SD, Standard Deviation; APACHE II, Acute Physiology and Chronic Health Evaluation II; TISS, Therapeutic intervention scoring system; FiO2, the fraction of inspired oxygen; PEEP, positive end-expiratory pressure; RR, respiratory rate; Mv, minute ventilation; PSL, pressure support level; PSLvolume, tidal volume with pressure support; Ppeak, peak inspiratory pressure; mPaw, mean airway pressure; SpO2: BT, body temperature; HR: heart rate; SBP, systolic blood pressure; DBP, diastolic blood pressure; GCS_E: Glasgow Coma Scale eye-opening; GCS_M, Glasgow Coma Scale-motor response; SBT, spontaneous breathing trials.

**FIGURE 3:**
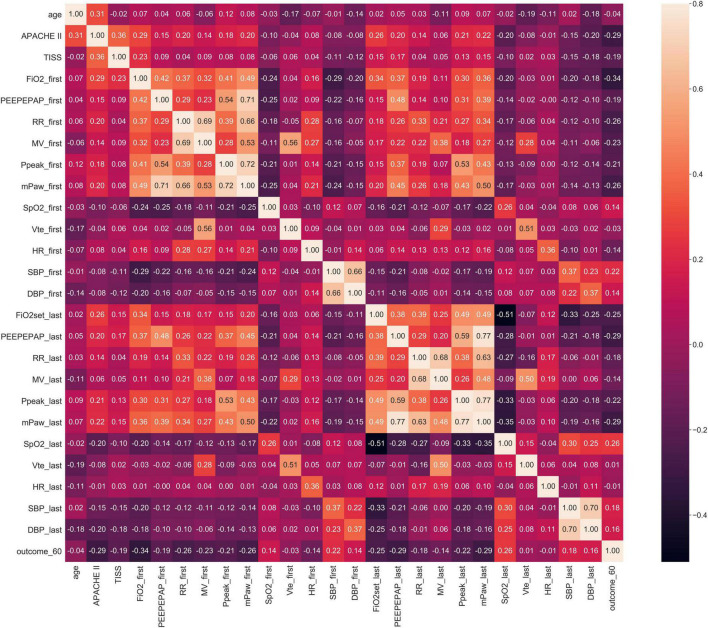
Stage 1 Spearman correlation (the 60th hour model). Note: outcome_60: Successful weaning or not before using 60 h of MV; FiO2_first, _last: the first/last value of FiO2 after using MV within 60 h, others are labeled similar.

**FIGURE 4:**
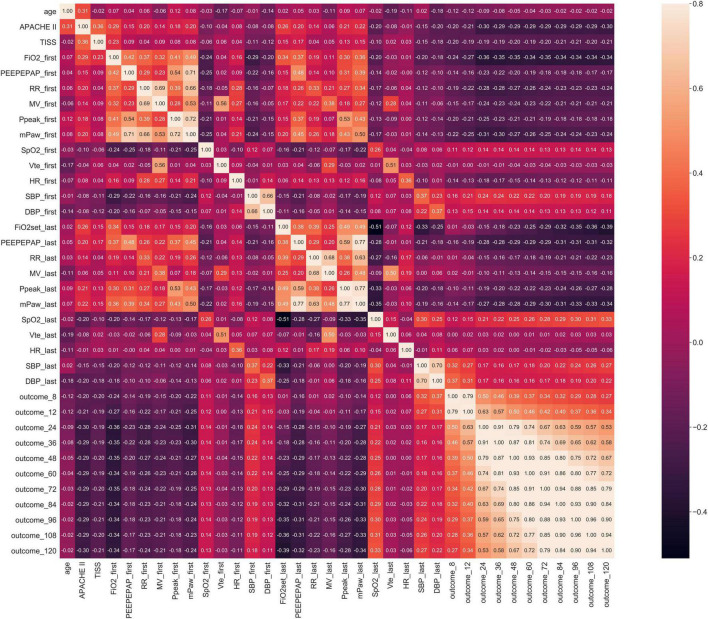
Stage 1 Spearman correlation.

**FIGURE 5:**
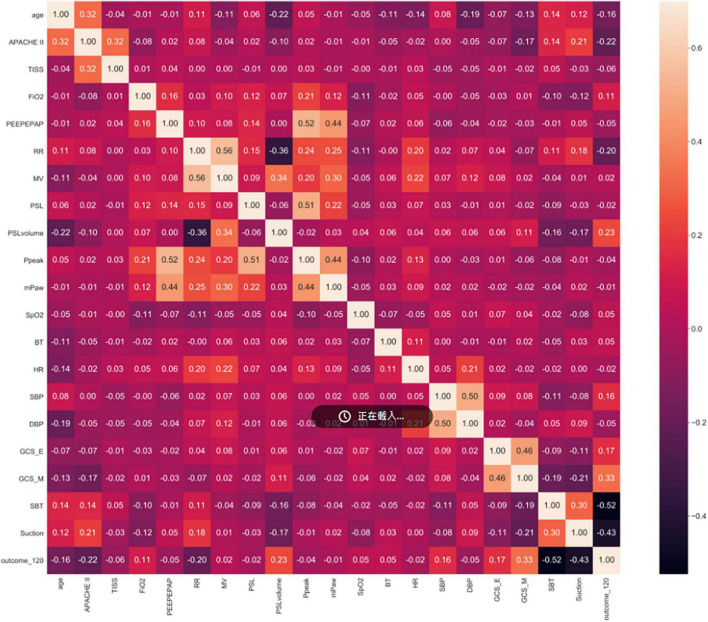
Stage 2 Spearman correlation (the 120th Hour model). Note: outcome_120: Successful weaning or not before using 120 h of MV, others are labeled similar.

**FIGURE 6:**
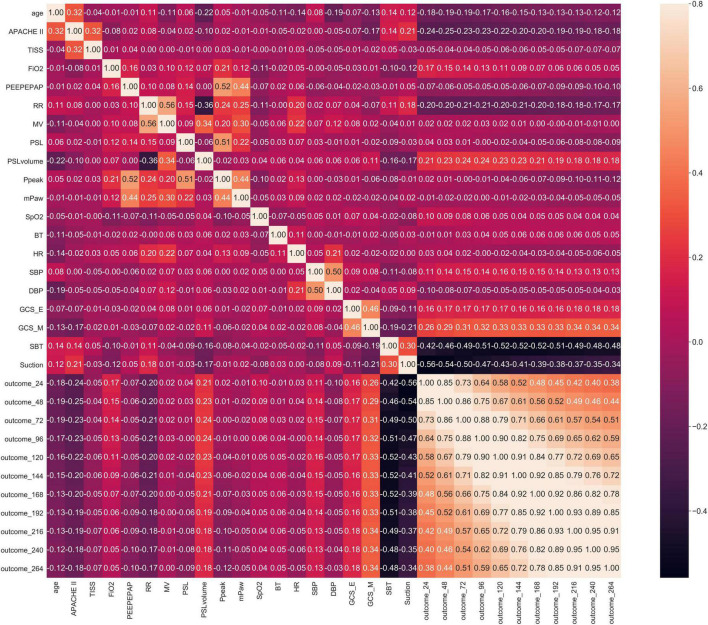
Stage 2 Spearman correlation.

For example in Stage 1, Spearman correlation analysis for the 60th hour model (Figure 3) showed that the most relevant to the timing of successful try-weaning was the first FiO_2_, followed by APACHE II score, and the last PEEP and mPaw. Spearman correlation analyses for all models in Stage 1 are shown in Figure 4. Moreover, for Stage 2, Spearman correlation analysis for the 120*^th^* hour model (Figure 5) showed that the number of SBTs was most relevant to the timing of successful weaning-MV, followed by the number of Suctions. Spearman correlation analyses for all models in Stage 2 are shown in Figure 6.

### Modeling results

**FIGURE 7:**
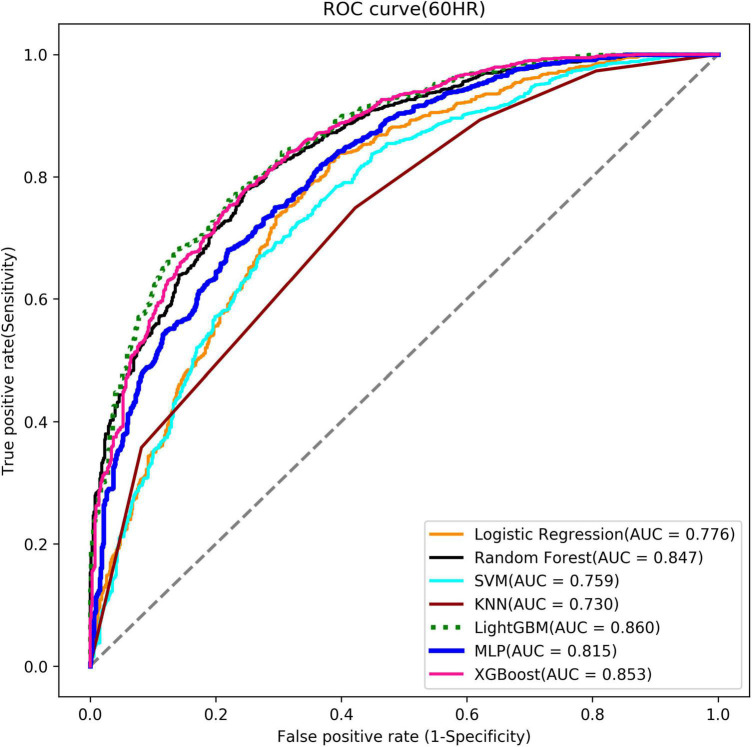
Stage 1 ROC curve (the 60th Hour model).

In this study, eleven models were established in each of the two stages. In Stage 1, the 60th-hour model was taken as an example. Each model used seven algorithms with optimal hyper-parameters. Models’ performances with the seven ML algorithms are shown in Table 3 (Stage 1) (Supplementary Table 1 for other models in Stage 1). With the 60*^th^*-hour model as an example, according to the value of AUC model, the lightGBM model obtained the maximum value (AUC = 0.860) and was used as the basis for implementing the online prediction system. Besides, ROC curve is a performance measurement for a classification model at various thresholds. Figure 7 covers the ROC curves of the seven algorithms and the three highest AUCs (lightGBM, XGBoost and Random forest) ranged from 0.860 to 0.847 showing good model quality with smooth empirical ROC curves and AUCs near to 1.

**TABLE 3:** Testing results of the predictive models: Stage 1 try-weaning model of the 60th HR and Stage 2 MV-weaning model of the 120th HR.

Algorithm	Accuracy	Sensitivity	Specificity	AUC
**Stage 1**				
Logistic regression	0.710	0.710	0.710	0.776
Random forest	0.760	0.760	0.760	0.847
SVM	0.716	0.778	0.609	0.759
KNN	0.686	0.749	0.578	0.730
LightGBM	0.768	0.788	0.733	0.860
MLP	0.732	0.746	0.709	0.815
XGBoost	0.774	0.806	0.718	0.853
**Stage 2**				
Logistic regression	0.827	0.827	0.826	0.913
Random forest	0.824	0.822	0.829	0.918
SVM	0.713	0.714	0.712	0.797
KNN	0.649	0.679	0.580	0.683
lightGBM	0.842	0.842	0.842	0.923
MLP	0.805	0.804	0.807	0.905
XGBoost	0.810	0.810	0.810	0.908

**FIGURE 8:**
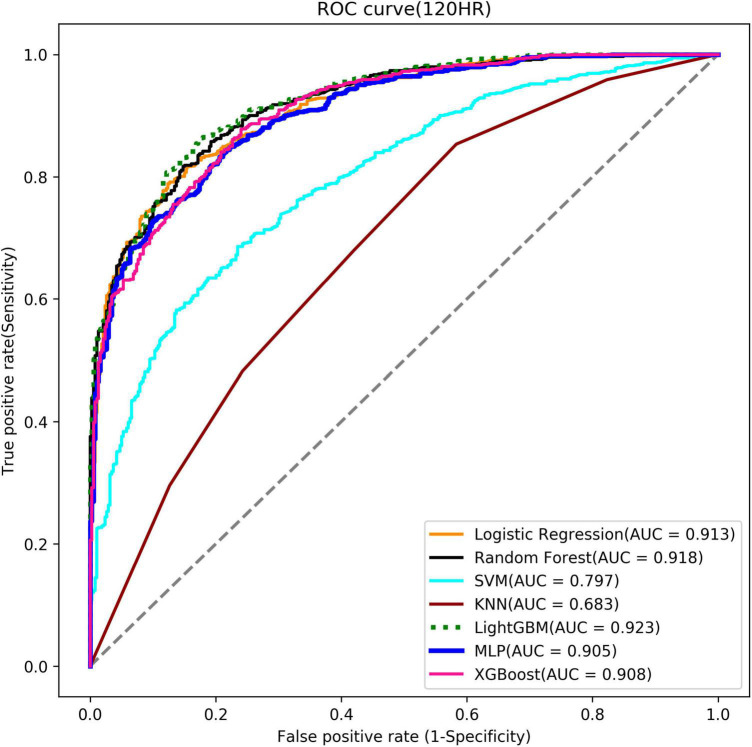
Stage 2 ROC curve (the 120th Hour model).

In Stage 2, the 120th hour (5th day) model was taken as an example. The lightGBM model was selected for implementation based on the AUCs of the seven algorithms (AUC = 0.923) [Table 3 (Stage 2)] (Supplementary Table 2 for other models in Stage 2). Figure 8 shows the ROC curves of the seven algorithms and the three highest AUCs (lightGBM, Random forest and Logistic regression) ranged from 0.913 to 0.923. It also shows excellent models. Hyper-parameters used for building optimal model for each algorithm are listed in Supplementary Table 3.

Moreover, we randomly chose patients A, B, and C who successfully weaned from MV in 2021 (weaning time points at the 144th hr, 216th hr, and 242th hr, respectively) and observed them retrospectively. Taking the data at the 48th-hour ventilator use as features (the patients all failed to wean at the 48th hr), the probabilities predicted by our 48th-hr model were all <50%, which mean a tendency for unsuccessful weaning (probabilities were 32.58, 40.24, and 20.1%, respectively). These predictions were correct. We then fed the same data to a single model (usually the last model, represented here by our 264th-hr model) and all displayed a tendency for successful weaning (probabilities were 95.23, 79.06, and 61.38%). These predictions were incorrect. This proves that, adopting in practical, using multiple models is more appropriate to the prediction of weaning time than when using a single model only.

### Prediction system development and deployment

Using the optimal prediction models, the AI Center and the Department of Information Systems of CMMC jointly developed the timing prediction system (a dashboard) for try-weaning and weaning MV and integrated it with the existing hospital information system (respiratory care system). Such graphical presentation and drill-down interactive function help track the status of patients and enhance users’ acceptance of the AI dashboard. Our results showed that this system could predict the optimal timing for try-weaning and weaning MV during the decision-making process of the clinicians. Moreover, the reference data from this system could be used effectively for communication with the patient’s family.

### Use case scenario

**FIGURE 9:**
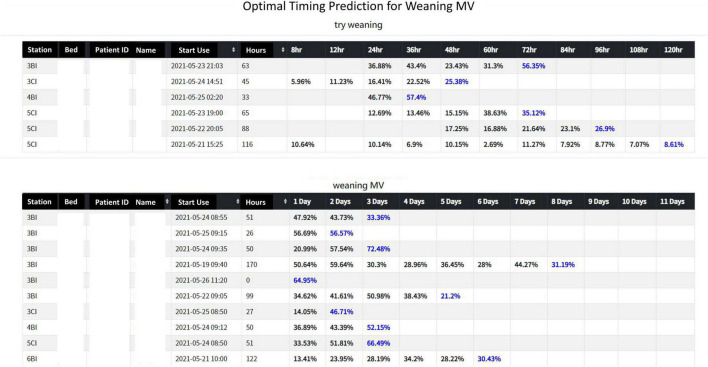
A screenshot of the artificial intelligence (AI) prediction system interface.

The interface of the AI dashboard is shown in Figure 9. Stage 1 (try-weaning) displayed the patient’s basic information (bed number, medical record number, name), the time when ventilator use was started, the current number of hours of use, and the probability of success in each period. For example, the first patient of Stage 1 had used the ventilator for 63 h; the system captured the patient’s characteristic data and displayed the predictions for the nearest future. It could be seen that the probability of successful try-weaning within 72 h of this patient was 56.35%, which implies that the medical team may switch the mode of the patient’s ventilator (start try-weaning) during this time. Stage 2 (weaning MV) presented content similar to Stage 1, which included basic information, starting time of support mode, current total hours of support mode, and the success probability of each period. For example, the first patient in Stage 2 had been in the support model for 51 h; the system predicted that the success probability of liberating the patient in MV within three days (72 h) was 33.36%, so it was not recommended to wean during this period.

### User evaluation and impact analysis of artificial intelligence assistance

After the hospital launched the dashboard system and implemented it for one month, we interviewed some of the respiratory care members (3 physicians and 5 therapists) and gained high positive feedback. They thought that the dashboard was a very useful tool in helping them determine the optimal timing for trying to wean a patient from the ventilator. According to them, it was also a useful tool for shared decision-making (SDM) especially when communicating with patients or their families. Also, they raised expectations for improvement. For example, they hoped that the predicted value at each time point could be drawn as a polyline to easily see the trend of the predicted probabilities for a patient. These expectations were later realized.

So far, this AI dashboard has been online in ICU for nearly two years. Therefore, we conducted an anonymous 5-scaled questionnaire survey (with Google Form) for all 10 ICU physicians during September 30, 2022 and October 5, 2022, and received 8 valid questionnaires. Overall, they believe that the AI is easy to use (mean = 4.5), the prediction results provided by the AI are of reference value (mean = 4.0), and the AI is helpful to the MV weaning decision (mean = 4.25). However, 2 physicians answered “seldom use AI”, 4 physicians answered “frequent use of AI”, and the remaining 2 physicians answered “already use AI regularly”. One of the physicians who answered “seldom use of AI” left a comment saying that physicians have had extensive experience in assessing MV weaning and AI assistance is not very necessary.

Moreover, we selected an ICU ward and recorded the successful extubation time and associated data. The collected data was then compared with that of the previous year. In other words, the parameters collected from July to November 2019 (without AI assistance) was contrasted with those of July to November 2020 (with AI assistance). Intubated adult patients weaned from MV successfully were enrolled in the study implementation. Patients with tracheostomy and transferred to the respiratory care ward were excluded. The analysis results of Table 4A showed no statistically significant difference in the demographic distribution between these two groups, including the age, gender, and disease severity (Apache II, TISS, COMA) of patients. It provided a fair basis for AI intervention comparison. It also showed that there was no significant difference in successful extubation-rate, indicating that patient safety was not compromised (actually slightly improved) with AI. However, in Table 4B, we noticed the average intubation hours after AI intervention were about 21 hours shorter than that without AI intervention, and the average stay in ICU was reduced by 0.5 days, showing that our AI-assisted system does boost patients wean from ventilators earlier, which could improve the quality of care.

**TABLE 4:** The results of clinical evaluation and comparison.

Feature	Overall	2019/07-11 (without AI)	2020/07-11 (with AI)	*P*-Value
		
	*N* = 171	*N* = 78	*N* = 93	
**(A) Analysis of weaning rate**				
Age, mean ± SD	66.2 ± 15.7	65.7 ± 16.3	66.7 ± 15.2	0.695
**Gender**				
Female, *n* (%)	58 (33.9)	29 (37.2)	29 (31.2)	0.507
Male, *n* (%)	113 (66.1)	49 (62.8)	64 (68.8)	
APACHE II score, mean ± SD	20.8 ± 8.5	20.3 ± 8.7	21.7 ± .5	0.262
TISS, mean ± SD	30.6 ± 6.5	30.1 ± 6.8	31.0 ± 6.4	0.243
COMA scale, mean ± SD	8.7 ± 3.5	9.1 ± 3.6	8.3 ± 3.4	0.068
**Extubation**				
Successful, *n* (%)	167 (97.7)	76 (97.4)	91 (97.8)	1.000
Failure, *n* (%)	4 (2.3)	2 (2.6)	2 (2.2)	

**Feature**	**Overall**	**2019/07-11 (without AI)**	**2020/07-11 (with AI)**	***P*-Value**
		
	***N* = 167**	***N* = 76**	***N* = 91**	

**(B) Analysis of successful weaning use-time**				
Age, mean ± SD	66.0 ± 15.8	65.7 ± 16.4	66.4 ± 15.1	0.814
**Gender**				
Female, *n* (%)	56 (33.5)	29 (38.2)	27 (29.7)	0.321
Male, *n* (%)	111 (66.5)	47 (61.8)	64 (70.3)	
APACHE II score, mean ± SD	20.9 ± 8.5	20.4 ± 8.8	21.8 ± 8.5	0.278
TISS, mean ± SD	30.7 ± 6.5	30.3 ± 6.8	31.1 ± 6.4	0.318
COMA scale, mean ± SD	8.6 ± 3.5	9.0 ± 3.5	8.3 ± 3.5	0.099
Intubation hours, mean ± SD	170.9 ± 150.7	178.0 ± 147.7	156.6 ± 150.4	0.300
ICU Days, mean ± SD	9.3 ± 7.5	9.3 ± 8.0	8.8 ± 6.9	0.631

We also performed Kappa analysis (P < 0.05 for significance) on the patients with AI to estimate the consistency of AI prediction and regular care procedure. As shown in Table 5, all values of Kappa are above 0.61 indicating that all models have substantial consistency between these two decision-making strategies (48).

**TABLE 5:** Analysis of Kappa values in 11 models of Stage 2.

Stage 2 model	Cohen Kappa
24 HR model	0.785
48 HR model	0.710
72 HR model	0.681
96 HR model	0.841
120 HR model	0.796
144 HR model	0.677
168 HR model	0.776
196 HR model	0.752
216 HR model	0.789
240 HR model	0.711
264 HR model	0.657

## Discussion

Most related studies in the past explored the factors that affect weaning from the ventilator or predicted the success of weaning. However, this study argues that precise weaning decisions should consist of two phases, try-weaning and complete weaning MV, and that each should have a separate predictive model built. In addition, we believe that, clinically, deciding on the optimal timing for weaning is more crucial than predicting the final success, so we built 11 models at 11 time points for each stage. More importantly, we used the optimal models to build a prediction system (AI dashboard) for monitoring all patients with MV in ICU to validate the feasibility of our comprehensive AI approach. The impact study confirmed that the average intubation time was shortened by 21 h after AI intervention. Overall, this study has significant academic and practical values.

Mechanical ventilation use is a life-guarding technique providing critically ill respiratory support, and it is one of the most common interventions given to ICU patients (49). In this study, correlation analyses for all models in Stage 1 showed that FiO2, mPaw, APACHE II score, PEEP, SpO2 tend to be higher correlated to the predictive models. It implies that oxygenation, hemodynamics and disease severity have great influence on full support mode shift to partial support mode. Increasing FiO2, mPaw and PEEP is to improve the patient’s oxygenation status, but too high mPaw and PEEP will cause lung overdistension and affect cardiac output. Similar, frequency of suction, numbers of SBT, GCS_M, APACHE II score, PSL volume, RR tend to higher correlate to the predictive models in Stage 2. It implies that cough strength, respiratory capacity and disease severity affected weaning success. This reminds clinical staff to assess the amount of sputum or secretions, the patient’s mobility, ability to cough, and breathing patterns to ensure successful extubation. However, the biomedical etiology and pathophysiology of weaning failure are complex and often multifactorial, including airway and lung dysfunction, brain dysfunction, cardiac dysfunction, diaphragm dysfunction, and endocrine dysfunction. Accordingly, determining the reason and subsequently developing a treatment strategy require a dedicated clinician with in-depth knowledge of these parameters of weaning failure (50). Moreover, earlier recognition of the patient’s capacity for some level of autonomous respiration is fundamental to progressively initiating the weaning of the patient from MV and finally gaining full independent respiratory function (51). Thus, our study provides a new AI-enabled solution to realize the expectation.

Ideally, the clinical weaning parameters collected in critical care need to be objective and easy to acquire, and the process would not impede patient management. The physiological mechanisms resulting in respiratory failure vary for different individuals, and diverse weaning parameters will contribute to one aspect of the pathophysiological mechanism. It has been proved that it is insufficient to improve the outcomes of ventilated patients by applying the weaning index only (52). Our AI models, which incorporate a full range of patients’ basic parameters, physiological parameters, and respiratory parameters, and consider the dynamic changes in time series, were used to establish a two-stage, multi-time series prediction model, which significantly improves the success in predicting weaning and conforms to clinical experience.

There have been several studies in the past that explored the prediction of related ICU respirator use with machine learning methods or traditional statistical methods (e.g., regression analysis method). Our research found that ML methods roughly outperformed traditional statistics. In the studies of ML method, we also obtained more excellent results. Our models are not only of high quality (AUC >0.94) but also the two-stage design is closer to clinical experience of weaning decision-making than a single-stage design. Cheng (53) also proposed a two-stage decision-making approach; however, our prediction model quality was more superior to theirs since we also subdivided each stage into 11 time points which helped to precisely grasp the timing of weaning MV and even shorten the intubation time. More importantly, among these studies, only our research realized the ML models in practice. We summarized the comparison of our study with previous works (53–56) in Table 6.

**TABLE 6:** A comparison with related studies.

Study	Patient group	Predictive outcome	ML algorithm (* best algorithm)	Sample size	Numbers of features	Model‘s performance (the highest AUC)	Real world implementation
**This Study**	Adult ICU patients with invasive mechanical ventilation	(1). Timing of full support shifting to partial support modes (2). Timing of weaning MV	Seven ML algorithms: LR, RF, SVM, KNN, LGBM, XGB, MLP. 11 models were established in each of the two stages. *The best algorithm: LGBM.	Stage 1: 5,873 Stage 2: 4,172	Stage 1: 25 Stage 2: 20	Stage 1: 0.843-0.953 Stage 2: 0.889-0.944	Yes. A predictive dashboard with best AI models was implemented and integrated into the existing HIS
(52)	Adult ICU patients with invasive mechanical ventilation	(1). The success shifting from full to partial support ventilation (2). Successful SBT	Seven ML algorithms: LR, Ridge Regression, Elastic Net, RF, SVM, ANN, XGB. 1 model was established in each of the two stages. *The best algorithm: XGB and RF.	First model: 2,153 Second model: 3,132	First model: 16 Second model: 12	First model: 0.76 Second model: 0.79	No
(53)	Cardiac Surgery patients with invasive mechanical ventilation	Successful weaned within 24 h	Six ML algorithms: LR, RF, SVM, DT, ANN, XGB. *The best algorithm: SVM.	1,439	28	0.88	No
(54)	Adult ICU patients with invasive mechanical ventilation	Successful extubation	Three ML algorithm: RF, LGBM, XGB. *The best algorithms: LGBM.	117 (Total number of labeled was 12,268)	57	0.950	No
(55)	Adult ICU patients with invasive mechanical ventilation	Successful extubation	Six ML algorithms: CNN, ANN, LR, SVM, DT, RF *The best algorithm: CNN.	2,299	25	0.94	No

MV, Mechanical Ventilation; LR, Logistic Regression; RF, Random Forest; SVM, Support Vector Machines; KNN, K Nearest Neighbor; LGBM, lightGBM; XGB: XGBoost; MLP, Multilayer Perception; CNN, Convolutional Neural Network, ANN, Artificial Neural Network; DT: Decision Tree.

Furthermore, LightGBM models were noted with the highest AUC values amidst the seven ML algorithms, consistent with Chen et al. (57). Moreover, LightGBM has been regarded as the most effective model to predict extubation success when compared with XGBoost, MLP, and SVM. LightGBM is a gradient boosting framework of tree-based learning algorithms with faster training speed and better accuracy, but with lower memory usage.

Further analysis indicated that our models had convinced predictability regarding the Swets classification (0.5 ≤ AUC ≤ 0.7, lower predicted; 0.7 ≤ AUC ≤ 0.9, certain predictive ability; AUC > 0.9, high predictive ability) (58). However, it was found that the models over time have a tendency of decreasing AUC (0.953∼0.864, lightGBM models in Stage 1; 0.943∼916, lightGBM models in Stage 2), which may imply that the longer the patient uses the ventilator, the more complicated it becomes when considering whether the patient can undergo try-weaning. This finding can also support why we use multiple periods instead of single period to predict weaning MV.

Our AI system could allow the clinicians to grasp the appropriate weaning time precisely, which could prevent the worthless dangers due to delayed or premature weaning process. Thus, with our AI system, the risks of complications and medical costs related to ventilatory support for patients are expected to decrease. More importantly, our AI system could lessen the effect of inter-clinician variability and improve the overall ICU care quality.

The deficiency of thorough evidence and the difference of results between individuals and subpopulations demonstrates there is scanty consensus on the issue of the best weaning protocol in clinical literature (59, 60). Our research results could provide useful solution to this long-standing clinical difficulty. AI applications should be aptly weighed parallel to other information sources and certified by well-designed prospective studies before comprehensive implementation. Although we noticed a substantial and even almost perfect consistency in the prediction of successful weaning from ventilators after AI intervention, we position our AI system as an auxiliary, not as a determiner for diagnosis.

Clinical decision assistance systems could aid clinicians in their decision-making (61) and provide individualized management protocols based on the patients’ clinical data and updated knowledge (62). Besides, AI is a powerful instrument that lowers the medical error rate and improves healthcare consistency and efficacy (63). However, there has been a lot of concern about the demerits of AI model applications in the decision of MV weaning. First, deep learning lacks explanatory power and related potential bias is hard to identify (64). Moreover, new ethical issues have been presented such as issues of erroneous decisions by AI, legal responsibility, and private information security crisis are taken into consideration (65).

There are limitations to our study. First, it is a single-center study, and we do not have an external cohort to validate our obtained models despite using data routinely collected in a real-world setting. Thus, extra care in terms of research generality must be given when extrapolating the findings to other centers. Second, some weaning-relevant data, like rehabilitation program arrangement, were not assessed in our dataset. We consider the model’s accuracy could be improved significantly after assessing this detailed information. Third, our enrolled patient number was relatively small, impacting the result. Fourth, our study failed to include essential features and modalities, like chest X-ray images, cuff-leak test, diaphragm ultrasonography, and fluid balance, which are widely assessed to predict successful extubation. Further, no information related to laryngeal edema after extubation was trained in our models. Therefore, it could be difficult for the developed model to forecast the extubation failure rate due to post-extubation laryngeal edema.

## Conclusion

Weaning timing assessment in ICU patients with MV is one of the most critical steps for respiratory care teams. We employed AI technology to develop a comprehensive system and embedded it into the existing HIS to predict the timing of weaning MV; this proves the clinical innovation of AI intervention in critical care. According to our knowledge, such a study with valuable academic and practical implications is rare.

Most studies only report the quality of predictive models; thus, it may be difficult to judge its actual clinical value. Our study established a predictive model and validated the model in the clinical field, which proved that it has better benefits than traditional ones. Therefore, our study supports that AI could be a promising approach in predicting MV weaning timing in ICU and is expected to advance clinical research in this field.

Although we can see that the AI prediction dashboard we proposed can be an effective tool to assist weaning decision-making, it should be noted that it cannot be regarded as the only dependence for final decision-making. That is, after referring to the AI’s prediction, the medical team still need to conduct and discuss a professional and comprehensive observation and evaluation of the patient again before making the final weaning decision.

Our study showed that the use of ML approaches could obtain better predictive ability in ICU, however, some physicians also reported that AI assistance is not very necessary. Thus, how to increase physicians’ willingness to accept AI is indeed a key research topic. Besides, AI algorithms are difficult to understand (so-called black-box), which may affect the trust of clinical staff. Therefore, follow-up research to improve the explainability of AI must be done. Furthermore, intensivists expect that AI can be applied to build a decision support tool for integrated consideration of a patient rather than simply providing predictions on an illness. This is a challenge that should be taken seriously. However, we still have a long way to go at this moment.

## Data availability statement

The original contributions presented in the study are included in the article/Supplementary material, further inquiries can be directed to the corresponding author/s.

## Ethics statement

The studies involving human participants were reviewed and approved by Chi Mei Medical Center (IRB Serial No.: 10912-016). Written informed consent for participation was not required for this study in accordance with the national legislation and the institutional requirements.

## Author contributions

C-MChen, K-CC, C-FL, and C-CC: conceptualization. C-MH, J-JW, C-CL, and C-CC: data curation. C-FL, M-IS, C-JC, and C-CC: formal analysis. C-MH, C-MChen, and C-CC: investigation. C-FL, M-IS, and C-JC: methodology. C-CL and C-MChen: project administration. S-CK, K-CC, M-IS, S-CH, and C-MChen: resources. C-FL and C-JC: software. C-CC: supervision. C-MH, S-CK, C-MChao, and C-CC: validation. C-MChao and C-MChen: visualization. C-FL and C-CC: writing—original draft. C-CC: writing—review and editing. All authors read and approved the submitted version.
