# Supplementary material for: An artificial intelligence system to predict the optimal timing for mechanical ventilation weaning for intensive care unit patients: A two-stage prediction approach
Source: Front Med (Lausanne). 2022 Nov 18;9:935366. doi: 10.3389/fmed.2022.935366 (PMC9715756; doi:10.3389/fmed.2022.935366)
Supplement: Supplementary file 1 [file Table_1.pdf]

Supplementary Table 1. Stage 1 try weaning models

Model 8 HR	Testing Set Validation				5-fold Cross-Validation				
Algorithm	Accuracy	Sensitivity	Specificity	AUC	Accuracy	Sensitivity	Specificity	AUC	AUC(95%CI)
Logistic Regression	0.826	0.805	0.831	0.893	0.817±0.009	0.827±0.010	0.815±0.010	0.821±0.009	0.801-0.841
Random Forest	0.851	0.850	0.852	0.938	0.919±0.005	0.767±0.011	0.955±0.007	0.861±0.004	0.851-0.871
SVM	0.814	0.796	0.818	0.878	0.800±0.012	0.842±0.013	0.791±0.015	0.816±0.009	0.795-0.838
KNN	0.780	0.856	0.763	0.868	0.813±0.007	0.819±0.015	0.812±0.009	0.816±0.008	0.798-0.833
LightGBM	0.884	0.880	0.884	0.953	0.938±0.006	0.784±0.013	0.974±0.006	0.879±0.008	0.862-0.896
MLP	0.888	0.859	0.894	0.941	0.901±0.019	0.809±0.030	0.922±0.025	0.865±0.016	0.829-0.902
XGboost	0.884	0.883	0.884	0.951	0.941±0.007	0.795±0.019	0.975±0.007	0.885±0.011	0.861-0.909

Model 12 HR	Testing Set Validation				5-fold Cross-Validation				
Algorithm	Accuracy	Sensitivity	Specificity	AUC	Accuracy	Sensitivity	Specificity	AUC	AUC(95%CI)
Logistic Regression	0.730	0.750	0.723	0.815	0.742±0.015	0.756±0.026	0.736±0.016	0.746±0.017	0.707-0.785
Random Forest	0.779	0.807	0.769	0.880	0.842±0.015	0.659±0.017	0.909±0.017	0.784±0.014	0.752-0.817
SVM	0.721	0.725	0.720	0.798	0.727±0.009	0.754±0.012	0.717±0.014	0.735±0.007	0.720-0.751
KNN	0.704	0.779	0.676	0.788	0.739±0.012	0.698±0.037	0.754±0.012	0.726±0.019	0.684-0.768
LightGBM	0.813	0.813	0.813	0.907	0.866±0.006	0.655±0.016	0.944±0.003	0.800±0.009	0.780-0.819
MLP	0.767	0.767	0.768	0.869	0.832±0.010	0.685±0.025	0.886±0.021	0.786±0.005	0.773-0.798
XGboost	0.801	0.800	0.801	0.897	0.863±0.003	0.659±0.013	0.939±0.005	0.799±0.005	0.788-0.811

Model 24 HR	Testing Set Validation				5-fold Cross-Validation				
Algorithm	Accuracy	Sensitivity	Specificity	AUC	Accuracy	Sensitivity	Specificity	AUC	AUC(95%CI)
Logistic Regression	0.730	0.730	0.730	0.796	0.737±0.006	0.792±0.018	0.686±0.019	0.739±0.005	0.727-0.751
Random Forest	0.772	0.800	0.745	0.857	0.778±0.009	0.814±0.008	0.744±0.023	0.779±0.009	0.759-0.800
SVM	0.722	0.722	0.722	0.786	0.720±0.015	0.857±0.013	0.592±0.039	0.724±0.015	0.691-0.758
KNN	0.669	0.756	0.588	0.743	0.695±0.016	0.671±0.021	0.717±0.021	0.694±0.016	0.658-0.730
LightGBM	0.820	0.823	0.818	0.893	0.804±0.010	0.808±0.018	0.800±0.010	0.804±0.011	0.780-0.828
MLP	0.758	0.761	0.756	0.849	0.758±0.006	0.713±0.053	0.801±0.054	0.757±0.006	0.743-0.771
XGboost	0.808	0.808	0.808	0.885	0.800±0.008	0.802±0.016	0.798±0.020	0.800±0.008	0.781-0.819

Model 36 HR	Testing Set Validation				5-fold Cross-Validation				
Algorithm	Accuracy	Sensitivity	Specificity	AUC	Accuracy	Sensitivity	Specificity	AUC	AUC(95%CI)
Logistic Regression	0.721	0.722	0.721	0.797	0.733±0.015	0.788±0.022	0.672±0.021	0.730±0.014	0.697-0.762
Random Forest	0.783	0.846	0.711	0.861	0.776±0.011	0.827±0.015	0.719±0.022	0.773±0.011	0.748-0.798
SVM	0.713	0.713	0.713	0.776	0.722±0.018	0.842±0.014	0.588±0.028	0.715±0.018	0.673-0.757
KNN	0.683	0.763	0.593	0.748	0.679±0.012	0.653±0.015	0.709±0.012	0.681±0.012	0.655-0.707
LightGBM	0.791	0.820	0.758	0.883	0.789±0.013	0.817±0.009	0.757±0.025	0.787±0.014	0.756-0.819
MLP	0.749	0.748	0.749	0.842	0.737±0.017	0.669±0.082	0.814±0.074	0.741±0.015	0.708-0.775
XGboost	0.795	0.825	0.760	0.873	0.784±0.008	0.808±0.013	0.756±0.017	0.782±0.008	0.764-0.800

Model 48 HR	Testing Set Validation				5-fold Cross-Validation				
Algorithm	Accuracy	Sensitivity	Specificity	AUC	Accuracy	Sensitivity	Specificity	AUC	AUC(95%CI)
Logistic Regression	0.701	0.700	0.702	0.775	0.738±0.008	0.786±0.008	0.666±0.016	0.726±0.009	0.706-0.745
Random Forest	0.754	0.754	0.755	0.839	0.773±0.006	0.846±0.011	0.663±0.022	0.755±0.008	0.737-0.773
SVM	0.709	0.710	0.709	0.770	0.735±0.006	0.841±0.007	0.575±0.017	0.708±0.007	0.692-0.725
KNN	0.677	0.746	0.573	0.723	0.657±0.014	0.629±0.018	0.698±0.016	0.664±0.014	0.632-0.695
LightGBM	0.777	0.777	0.777	0.861	0.792±0.008	0.856±0.009	0.695±0.018	0.776±0.009	0.756-0.796
MLP	0.736	0.736	0.735	0.820	0.731±0.021	0.708±0.081	0.765±0.070	0.737±0.007	0.721-0.752
XGboost	0.763	0.763	0.763	0.848	0.788±0.004	0.851±0.006	0.692±0.013	0.772±0.005	0.761-0.783

Model 60 HR	Testing Set Validation				5-fold Cross-Validation				
Algorithm	Accuracy	Sensitivity	Specificity	AUC	Accuracy	Sensitivity	Specificity	AUC	AUC(95%CI)
Logistic Regression	0.710	0.710	0.710	0.776	0.738±0.013	0.779±0.016	0.667±0.010	0.723±0.013	0.695-0.752
Random Forest	0.760	0.760	0.760	0.847	0.778±0.010	0.855±0.004	0.646±0.021	0.751±0.012	0.723-0.778
SVM	0.716	0.778	0.609	0.759	0.731±0.019	0.829±0.012	0.564±0.038	0.697±0.022	0.647-0.747
KNN	0.686	0.749	0.578	0.730	0.642±0.014	0.615±0.013	0.690±0.019	0.652±0.015	0.619-0.685
LightGBM	0.768	0.788	0.733	0.860	0.793±0.007	0.870±0.010	0.662±0.010	0.766±0.007	0.750-0.782
MLP	0.732	0.746	0.709	0.815	0.733±0.022	0.742±0.053	0.716±0.037	0.729±0.013	0.700-0.758
XGboost	0.774	0.806	0.718	0.853	0.787±0.010	0.861±0.017	0.660±0.020	0.760±0.009	0.739-0.782

Model 72 HR	Testing Set Validation				5-fold Cross-Validation				
Algorithm	Accuracy	Sensitivity	Specificity	AUC	Accuracy	Sensitivity	Specificity	AUC	AUC(95%CI)
Logistic Regression	0.739	0.790	0.633	0.775	0.739±0.011	0.781±0.017	0.652±0.030	0.716±0.013	0.687-0.746
Random Forest	0.747	0.761	0.720	0.829	0.781±0.005	0.865±0.011	0.607±0.023	0.736±0.008	0.718-0.754
SVM	0.726	0.781	0.614	0.759	0.748±0.009	0.848±0.008	0.539±0.035	0.693±0.015	0.659-0.728
KNN	0.663	0.691	0.607	0.706	0.635±0.012	0.616±0.018	0.675±0.027	0.645±0.013	0.615-0.675
LightGBM	0.761	0.761	0.760	0.848	0.799±0.008	0.894±0.005	0.602±0.021	0.748±0.011	0.722-0.774
MLP	0.737	0.732	0.747	0.817	0.722±0.038	0.718±0.080	0.729±0.056	0.724±0.017	0.684-0.763
XGboost	0.741	0.741	0.740	0.828	0.789±0.008	0.874±0.007	0.612±0.014	0.743±0.009	0.723-0.763

Model 84 HR	Testing Set Validation				5-fold Cross-Validation				
Algorithm	Accuracy	Sensitivity	Specificity	AUC	Accuracy	Sensitivity	Specificity	AUC	AUC(95%CI)
Logistic Regression	0.712	0.712	0.712	0.775	0.748±0.017	0.784±0.025	0.662±0.024	0.723±0.014	0.691-0.755
Random Forest	0.765	0.784	0.720	0.827	0.796±0.004	0.873±0.012	0.614±0.024	0.744±0.007	0.727-0.761
SVM	0.723	0.781	0.587	0.737	0.755±0.010	0.850±0.024	0.531±0.025	0.691±0.004	0.681-0.700
KNN	0.660	0.699	0.568	0.686	0.634±0.011	0.622±0.013	0.663±0.028	0.643±0.014	0.611-0.674
LightGBM	0.751	0.750	0.752	0.842	0.805±0.008	0.904±0.008	0.574±0.026	0.739±0.012	0.711-0.767
MLP	0.717	0.716	0.718	0.799	0.739±0.027	0.762±0.060	0.687±0.053	0.725±0.008	0.706-0.743
XGboost	0.762	0.763	0.759	0.844	0.806±0.012	0.898±0.015	0.588±0.031	0.743±0.015	0.708-0.778

Model 96 HR	Testing Set Validation				5-fold Cross-Validation				
Algorithm	Accuracy	Sensitivity	Specificity	AUC	Accuracy	Sensitivity	Specificity	AUC	AUC(95%CI)
Logistic Regression	0.711	0.711	0.711	0.781	0.753±0.010	0.789±0.014	0.657±0.032	0.723±0.014	0.691-0.755
Random Forest	0.740	0.740	0.741	0.821	0.807±0.009	0.883±0.007	0.606±0.046	0.744±0.020	0.698-0.790
SVM	0.707	0.706	0.707	0.771	0.770±0.007	0.856±0.009	0.537±0.024	0.697±0.011	0.672-0.722
KNN	0.671	0.717	0.550	0.700	0.637±0.011	0.626±0.023	0.667±0.033	0.647±0.010	0.624-0.670
LightGBM	0.752	0.752	0.751	0.843	0.823±0.012	0.927±0.011	0.542±0.051	0.735±0.023	0.683-0.787
MLP	0.740	0.739	0.741	0.824	0.711±0.041	0.714±0.087	0.703±0.096	0.709±0.018	0.668-0.749
XGboost	0.762	0.783	0.705	0.836	0.822±0.011	0.919±0.018	0.560±0.054	0.740±0.022	0.691-0.788

Model 108 HR	Testing Set Validation				5-fold Cross-Validation				
Algorithm	Accuracy	Sensitivity	Specificity	AUC	Accuracy	Sensitivity	Specificity	AUC	AUC(95%CI)
Logistic Regression	0.743	0.783	0.629	0.785	0.754±0.013	0.788±0.022	0.654±0.034	0.721±0.014	0.689-0.752
Random Forest	0.766	0.782	0.718	0.842	0.812±0.012	0.884±0.017	0.599±0.029	0.742±0.014	0.711-0.772
SVM	0.736	0.789	0.580	0.748	0.777±0.011	0.860±0.013	0.537±0.025	0.698±0.014	0.668-0.729
KNN	0.666	0.693	0.587	0.696	0.644±0.017	0.630±0.014	0.686±0.026	0.658±0.019	0.614-0.702
LightGBM	0.755	0.755	0.753	0.846	0.832±0.010	0.937±0.009	0.527±0.025	0.732±0.014	0.701-0.763
MLP	0.747	0.764	0.698	0.813	0.743±0.031	0.763±0.065	0.684±0.075	0.723±0.013	0.695-0.752
XGboost	0.761	0.777	0.713	0.829	0.824±0.010	0.923±0.007	0.537±0.031	0.730±0.017	0.692-0.768

Model 120 HR	Testing Set Validation				5-fold Cross-Validation				
Algorithm	Accuracy	Sensitivity	Specificity	AUC	Accuracy	Sensitivity	Specificity	AUC	AUC(95%CI)
Logistic Regression	0.733	0.733	0.734	0.810	0.770±0.006	0.802±0.009	0.665±0.035	0.733±0.015	0.700-0.767
Random Forest	0.773	0.773	0.773	0.861	0.829±0.013	0.897±0.011	0.604±0.031	0.751±0.018	0.710-0.792
SVM	0.758	0.805	0.602	0.778	0.798±0.012	0.871±0.010	0.555±0.040	0.713±0.021	0.667-0.760
KNN	0.679	0.709	0.578	0.694	0.646±0.015	0.643±0.017	0.657±0.027	0.650±0.016	0.614-0.686
LightGBM	0.767	0.767	0.768	0.864	0.846±0.009	0.945±0.004	0.521±0.032	0.733±0.017	0.695-0.771
MLP	0.754	0.767	0.710	0.828	0.738±0.038	0.741±0.058	0.728±0.036	0.735±0.016	0.698-0.771
XGboost	0.763	0.763	0.763	0.856	0.842±0.009	0.937±0.005	0.530±0.031	0.734±0.017	0.696-0.771
