# Supplementary material for: An artificial intelligence system to predict the optimal timing for mechanical ventilation weaning for intensive care unit patients: A two-stage prediction approach
Source: Front Med (Lausanne). 2022 Nov 18;9:935366. doi: 10.3389/fmed.2022.935366 (PMC9715756; doi:10.3389/fmed.2022.935366)
Supplement: Supplementary file 2 [file Table_2.pdf]

Supplementary Table 2. Stage 2 weaning models

Model 24 HR	Testing Set Validation				5-fold Cross-Validation				
Algorithm	Accuracy	Sensitivity	Specificity	AUC	Accuracy	Sensitivity	Specificity	AUC	AUC(95%CI)
Logistic Regression	0.863	0.893	0.839	0.940	0.853±0.008	0.884±0.011	0.828±0.009	0.856±0.008	0.838-0.874
Random Forest	0.875	0.876	0.875	0.941	0.874±0.004	0.872±0.018	0.876±0.010	0.874±0.005	0.862-0.886
SVM	0.716	0.718	0.714	0.790	0.766±0.008	0.803±0.023	0.738±0.009	0.771±0.009	0.750-0.791
KNN	0.653	0.699	0.617	0.706	0.663±0.018	0.553±0.038	0.747±0.010	0.650±0.020	0.605-0.695
LightGBM	0.875	0.867	0.880	0.943	0.870±0.004	0.849±0.021	0.886±0.011	0.868±0.006	0.855-0.881
MLP	0.858	0.876	0.844	0.938	0.845±0.021	0.914±0.041	0.792±0.066	0.853±0.014	0.821-0.885
XGboost	0.865	0.860	0.869	0.935	0.832±0.011	0.680±0.022	0.949±0.009	0.814±0.012	0.788-0.841

Model 48 HR	Testing Set Validation				5-fold Cross-Validation				
Algorithm	Accuracy	Sensitivity	Specificity	AUC	Accuracy	Sensitivity	Specificity	AUC	AUC(95%CI)
Logistic Regression	0.875	0.892	0.858	0.940	0.851±0.016	0.872±0.029	0.830±0.014	0.851±0.016	0.815-0.886
Random Forest	0.869	0.869	0.869	0.944	0.860±0.008	0.849±0.021	0.872±0.007	0.861±0.008	0.842-0.879
SVM	0.731	0.730	0.732	0.806	0.764±0.029	0.780±0.038	0.747±0.020	0.764±0.028	0.700-0.828
KNN	0.668	0.698	0.636	0.714	0.651±0.026	0.553±0.021	0.753±0.031	0.653±0.026	0.594-0.712
LightGBM	0.870	0.870	0.869	0.944	0.857±0.005	0.850±0.017	0.864±0.012	0.857±0.005	0.846-0.868
MLP	0.867	0.870	0.864	0.939	0.851±0.012	0.854±0.046	0.847±0.049	0.851±0.012	0.824-0.877
XGboost	0.864	0.859	0.869	0.935	0.824±0.005	0.708±0.009	0.945±0.010	0.827±0.005	0.816-0.838

Model 72 HR	Testing Set Validation				5-fold Cross-Validation				
Algorithm	Accuracy	Sensitivity	Specificity	AUC	Accuracy	Sensitivity	Specificity	AUC	AUC(95%CI)
Logistic Regression	0.840	0.852	0.824	0.921	0.846±0.008	0.857±0.013	0.831±0.017	0.844±0.008	0.826-0.862
Random Forest	0.847	0.861	0.828	0.923	0.845±0.011	0.872±0.017	0.807±0.014	0.840±0.011	0.815-0.864
SVM	0.708	0.702	0.717	0.797	0.765±0.026	0.768±0.030	0.762±0.024	0.765±0.025	0.707-0.822
KNN	0.645	0.675	0.603	0.692	0.629±0.031	0.554±0.037	0.734±0.030	0.644±0.030	0.575-0.713
LightGBM	0.847	0.879	0.800	0.927	0.837±0.012	0.846±0.014	0.825±0.014	0.835±0.012	0.808-0.863
MLP	0.840	0.817	0.874	0.919	0.837±0.008	0.841±0.036	0.831±0.055	0.836±0.013	0.808-0.865
XGboost	0.834	0.833	0.835	0.922	0.812±0.012	0.726±0.017	0.935±0.015	0.831±0.012	0.803-0.858

Model 96 HR	Testing Set Validation				5-fold Cross-Validation				
Algorithm	Accuracy	Sensitivity	Specificity	AUC	Accuracy	Sensitivity	Specificity	AUC	AUC(95%CI)
Logistic Regression	0.827	0.828	0.827	0.918	0.826±0.013	0.836±0.022	0.808±0.031	0.822±0.014	0.791-0.853
Random Forest	0.830	0.829	0.831	0.919	0.843±0.011	0.876±0.023	0.781±0.022	0.829±0.008	0.810-0.847
SVM	0.732	0.728	0.740	0.815	0.756±0.013	0.749±0.023	0.768±0.022	0.758±0.011	0.734-0.783
KNN	0.671	0.685	0.645	0.710	0.621±0.015	0.564±0.027	0.727±0.019	0.645±0.012	0.618-0.673
LightGBM	0.841	0.840	0.843	0.928	0.839±0.012	0.869±0.024	0.783±0.024	0.826±0.009	0.806-0.847
MLP	0.824	0.836	0.802	0.916	0.820±0.017	0.794±0.053	0.868±0.055	0.831±0.009	0.810-0.852
XGboost	0.827	0.828	0.827	0.915	0.809±0.019	0.756±0.035	0.906±0.011	0.831±0.013	0.803-0.860

Model 120 HR	Testing Set Validation				5-fold Cross-Validation				
Algorithm	Accuracy	Sensitivity	Specificity	AUC	Accuracy	Sensitivity	Specificity	AUC	AUC(95%CI)
Logistic Regression	0.827	0.827	0.826	0.913	0.826±0.020	0.832±0.017	0.811±0.032	0.821±0.023	0.770-0.873
Random Forest	0.824	0.822	0.829	0.918	0.841±0.016	0.878±0.016	0.758±0.027	0.818±0.017	0.778-0.857
SVM	0.713	0.714	0.712	0.797	0.754±0.012	0.744±0.021	0.777±0.028	0.760±0.012	0.733-0.788
KNN	0.649	0.679	0.580	0.683	0.620±0.016	0.584±0.024	0.705±0.024	0.644±0.014	0.614-0.675
LightGBM	0.842	0.842	0.842	0.923	0.835±0.011	0.887±0.012	0.716±0.008	0.801±0.010	0.778-0.824
MLP	0.805	0.804	0.807	0.905	0.821±0.013	0.837±0.032	0.785±0.059	0.811±0.019	0.768-0.854
XGboost	0.810	0.810	0.810	0.908	0.810±0.013	0.787±0.019	0.865±0.009	0.826±0.011	0.802-0.850

Model 144 HR	Testing Set Validation				5-fold Cross-Validation				
Algorithm	Accuracy	Sensitivity	Specificity	AUC	Accuracy	Sensitivity	Specificity	AUC	AUC(95%CI)
Logistic Regression	0.818	0.826	0.795	0.902	0.818±0.014	0.824±0.015	0.802±0.031	0.813±0.018	0.773-0.853
Random Forest	0.812	0.813	0.810	0.902	0.837±0.007	0.879±0.013	0.722±0.047	0.801±0.019	0.758-0.843
SVM	0.723	0.723	0.722	0.800	0.755±0.013	0.740±0.008	0.795±0.034	0.768±0.019	0.725-0.810
KNN	0.664	0.691	0.589	0.687	0.618±0.017	0.589±0.021	0.697±0.032	0.643±0.018	0.602-0.685
LightGBM	0.812	0.817	0.801	0.898	0.850±0.011	0.921±0.012	0.652±0.061	0.786±0.026	0.728-0.845
MLP	0.800	0.801	0.795	0.894	0.796±0.015	0.787±0.039	0.821±0.053	0.804±0.009	0.785-0.824
XGboost	0.815	0.815	0.813	0.896	0.814±0.012	0.810±0.020	0.826±0.026	0.818±0.011	0.794-0.842

Model 168 HR	Testing Set Validation				5-fold Cross-Validation				
Algorithm	Accuracy	Sensitivity	Specificity	AUC	Accuracy	Sensitivity	Specificity	AUC	AUC(95%CI)
Logistic Regression	0.804	0.804	0.805	0.904	0.814±0.018	0.824±0.018	0.781±0.042	0.803±0.024	0.749-0.856
Random Forest	0.831	0.833	0.822	0.915	0.845±0.017	0.892±0.018	0.692±0.023	0.792±0.017	0.754-0.830
SVM	0.730	0.730	0.729	0.804	0.753±0.014	0.740±0.015	0.794±0.043	0.767±0.021	0.719-0.815
KNN	0.635	0.650	0.586	0.674	0.623±0.015	0.605±0.017	0.682±0.042	0.643±0.021	0.595-0.692
LightGBM	0.819	0.819	0.818	0.917	0.851±0.019	0.921±0.022	0.623±0.033	0.772±0.020	0.726-0.818
MLP	0.792	0.792	0.795	0.896	0.799±0.031	0.805±0.055	0.777±0.071	0.791±0.022	0.741-0.842
XGboost	0.819	0.823	0.805	0.904	0.831±0.016	0.848±0.018	0.776±0.026	0.812±0.017	0.773-0.851

Model 192 HR	Testing Set Validation				5-fold Cross-Validation				
Algorithm	Accuracy	Sensitivity	Specificity	AUC	Accuracy	Sensitivity	Specificity	AUC	AUC(95%CI)
Logistic Regression	0.787	0.778	0.822	0.890	0.808±0.008	0.816±0.013	0.778±0.033	0.797±0.014	0.766-0.828
Random Forest	0.804	0.804	0.802	0.889	0.849±0.004	0.891±0.005	0.685±0.019	0.788±0.009	0.768-0.808
SVM	0.730	0.730	0.729	0.811	0.744±0.010	0.730±0.016	0.798±0.019	0.764±0.008	0.747-0.781
KNN	0.671	0.701	0.554	0.685	0.637±0.009	0.630±0.006	0.663±0.024	0.647±0.015	0.614-0.680
LightGBM	0.823	0.822	0.826	0.911	0.864±0.009	0.931±0.009	0.607±0.034	0.769±0.017	0.731-0.807
MLP	0.804	0.804	0.802	0.887	0.771±0.009	0.758±0.017	0.821±0.039	0.790±0.014	0.759-0.821
XGboost	0.804	0.803	0.806	0.896	0.846±0.003	0.872±0.008	0.744±0.031	0.808±0.012	0.781-0.836

Model 216 HR	Testing Set Validation				5-fold Cross-Validation				
Algorithm	Accuracy	Sensitivity	Specificity	AUC	Accuracy	Sensitivity	Specificity	AUC	AUC(95%CI)
Logistic Regression	0.779	0.778	0.784	0.887	0.808±0.013	0.810±0.018	0.795±0.021	0.803±0.010	0.780-0.825
Random Forest	0.783	0.783	0.784	0.882	0.852±0.014	0.892±0.014	0.675±0.045	0.784±0.024	0.730-0.838
SVM	0.730	0.730	0.732	0.797	0.744±0.025	0.732±0.027	0.800±0.031	0.766±0.024	0.712-0.820
KNN	0.588	0.567	0.680	0.658	0.653±0.024	0.657±0.030	0.636±0.013	0.647±0.016	0.611-0.683
LightGBM	0.812	0.811	0.814	0.889	0.862±0.014	0.915±0.017	0.627±0.050	0.771±0.024	0.717-0.825
MLP	0.780	0.781	0.779	0.882	0.783±0.048	0.780±0.076	0.795±0.080	0.788±0.015	0.755-0.821
XGboost	0.769	0.770	0.766	0.870	0.852±0.016	0.883±0.021	0.718±0.030	0.800±0.016	0.764-0.837

Model 240 HR	Testing Set Validation	5-fold Cross-Validation

Algorithm	Accuracy	Sensitivity	Specificity	AUC	Accuracy	Sensitivity	Specificity	AUC	AUC(95%CI)
Logistic Regression	0.792	0.791	0.793	0.894	0.807±0.016	0.809±0.017	0.793±0.024	0.801±0.016	0.764-0.838
Random Forest	0.809	0.809	0.808	0.895	0.860±0.010	0.898±0.014	0.677±0.036	0.788±0.015	0.753-0.822
SVM	0.756	0.757	0.756	0.839	0.741±0.018	0.729±0.022	0.800±0.034	0.765±0.018	0.724-0.806
KNN	0.685	0.701	0.610	0.706	0.655±0.015	0.662±0.022	0.623±0.025	0.642±0.007	0.627-0.657
LightGBM	0.819	0.819	0.822	0.905	0.860±0.009	0.910±0.010	0.614±0.025	0.762±0.013	0.733-0.792
MLP	0.797	0.797	0.798	0.898	0.802±0.014	0.817±0.020	0.730±0.040	0.774±0.018	0.734-0.813
XGboost	0.808	0.808	0.808	0.902	0.865±0.013	0.896±0.014	0.710±0.032	0.803±0.017	0.764-0.842

Model 264 HR	Testing Set Validation				5-fold Cross-Validation				
Algorithm	Accuracy	Sensitivity	Specificity	AUC	Accuracy	Sensitivity	Specificity	AUC	AUC(95%CI)
Logistic Regression	0.826	0.826	0.827	0.912	0.811±0.015	0.815±0.015	0.789±0.026	0.802±0.019	0.759-0.845
Random Forest	0.836	0.836	0.837	0.910	0.864±0.010	0.901±0.013	0.661±0.029	0.781±0.014	0.750-0.812
SVM	0.753	0.753	0.755	0.821	0.746±0.005	0.738±0.008	0.790±0.022	0.764±0.009	0.744-0.785
KNN	0.740	0.815	0.337	0.655	0.673±0.011	0.682±0.014	0.625±0.017	0.654±0.010	0.632-0.675
LightGBM	0.832	0.832	0.832	0.916	0.870±0.014	0.914±0.013	0.630±0.042	0.772±0.024	0.718-0.826
MLP	0.820	0.822	0.811	0.898	0.794±0.012	0.805±0.026	0.739±0.080	0.739±0.080	0.707-0.836
XGboost	0.804	0.804	0.801	0.902	0.872±0.009	0.904±0.007	0.699±0.056	0.801±0.027	0.739-0.863
