# Supplementary material for: An artificial intelligence system to predict the optimal timing for mechanical ventilation weaning for intensive care unit patients: A two-stage prediction approach
Source: Front Med (Lausanne). 2022 Nov 18;9:935366. doi: 10.3389/fmed.2022.935366 (PMC9715756; doi:10.3389/fmed.2022.935366)
Supplement: Supplementary file 3 [file Table_3.pdf]

Supplementary Table 3. Hyper-parameters range for experiments

Method and Hyper-parameter	Outcomes stage1 8HR-120HR	Outcomes stage2 24HR-264HR
	Values	Values
Logistic regression		
penalty	None,l1, l2	None,l1, l2
C	1, 3,5	1,2, 3,5
max_iter	50,100,150	50,100,150
Random forest		
n_estimators	100, 300,500	100, 300,500
max_depth	5,6,7,8	5,6,7,8
min_samples_split	2, 5,10, 15	2, 4, 6, 10, 15
max_features	auto, sqrt,	auto, sqrt,
criterion	gini,entropy	gini,entropy
KNN		
n_neighbors	range(1,10)	range(1,10)
weights	uniform, distance	uniform, distance
algorithm	auto, ball_tree, kd_tree, brute	auto, ball_tree, kd_tree, brute
leaf_size	range(1,35)	range(1,35)
SVM		
kernel	rbf, linear	rbf, linear
gamma	scale, auto	scale, auto
C	1, 5,10	1, 5,10
random_state	None, range(12,32,42)	None, range(12,32,42)
decision_function_shape	ovo, ovr	ovo, ovr
shrinking	True, False	True, False
LightGBM		
learning_rate	0.001,0.01,0.1	0.001,0.01,0.1
num_iterations	100, 150,200	100, 150,200
max_depth	-1,1	-1,1
random_state	1, 17, 42	12, 32, 42
XGBoost		
learning_rate	1e-1,1e-2, 1e-3	1e-1,1e-2, 1e-3
gamma	1e-1,1e-2, 1e-3	1e-1,1e-2, 1e-3
num_iterations	100, 200, 300	100, 200, 300
max_depth	15, 25, 30, 50	15, 25, 30
num_parallel_tree	2, 5, 15	2, 5, 15
random_state	12, 32, 42	12, 32, 42
MLPClassifier		
hidden_layer_sizes	(250, 110, 75),<br>(128, 64, 32),(128,32)	(128, 64, 32),<br>(100, 50,),(128,32)
learning_rate_init	0.01, 0.001, 0.0001	0.01, 0.001, 0.0001
max_iter	100, 300, 500	100, 300, 500
batch_size	30, 50, 100	30, 50, 100
early_stopping	True, False	True, False

The hyper-parameters that are not described in this table are set to the default values used in the scikit-learn library
